# Psychology and technology: how Virtual Reality can boost psychotherapy and neurorehabilitation

**DOI:** 10.3934/Neuroscience.2022025

**Published:** 2022-11-14

**Authors:** C. M. Vicario, G. Martino

**Affiliations:** 1 COSPECS Department, University of Messina, 98122 Messina, Italy; 2 Department of Clinical and Experimental Medicine, University of Messina, 98122 Messina, Italy

**Keywords:** Virtual Reality, clinical psychology, clinical neuroscience, psychotherapy, neurorehabilitation

## 1. Introduction

**Figure 1.**
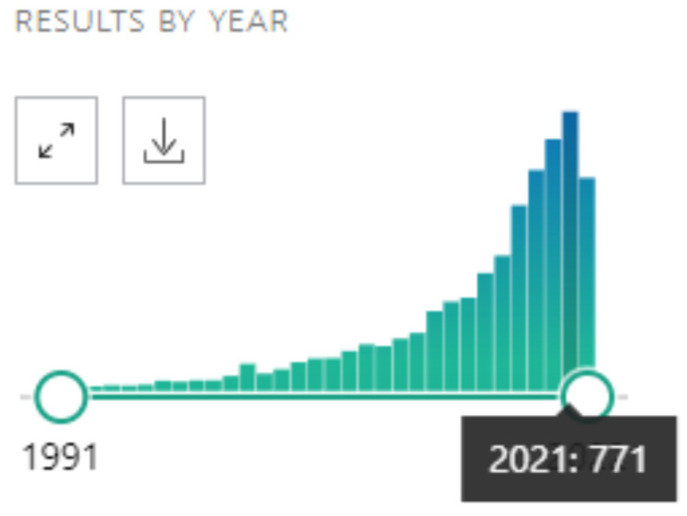
The year-by-year trend of publications in the field of psychology and virtual reality.

The use of the metaverse in the field of psychological research is constantly growing. This is easily verified by consulting PubMed, a free search engine of biomedical scientific literature online since 1996. By entering “psychology” and “virtual reality” (VR) as search keywords, it is possible to verify a progressive growth in the number of articles in the sector that in a few years it has gone from a few dozen to several hundred articles per year (Figure 1).

An important field of application of VR is clinical psychology. The use of computer screen-displayed clinically relevant scenarios for therapy has a limited impact in the field, due to the relatively low ecological validity of these experiences. VR technology offers a solution to this limitation by allowing the opportunity to actively perceiving one's own body within a simulated environment, letting individuals to experience reality-like situations [1]–[5] (Lucifora et al., 2020; Lucifora et al., 2021a; Daher et al., 2021; Grasso et al., 2020; Grasso et al., 2019). Furthermore, VR allows the creation of a safe and ecological environment within which it is possible to study behaviours that would otherwise not be easily assessed.

Talking about clinical practice, VR is becoming an increasingly established and widespread method alternative to in-vivo exposition for the treatments of several mental disorders. Recent studies have shown that this approach provides better results compared to traditional therapies for mental disorders [6] (Botella et al., 2017), including the treatment of Post-Traumatic Stress Disorder and several forms of phobias [7],[8] (Parsons and Rizzo, 2008; Anderson et al., 2005), and depression [9] (Baghaei et al., 2021). Importantly, VR offers promising benefits also for the treatment of paediatric disorders by reducing children's experience of aversive stimuli and anxiety levels [10] (Parson et al., 2017).

Advanced applications of VR for the treatment of anxiety and fear could refer to an integrated human-computer system [11] (Lucifora et al., 2021b), in which psychophysiological measures such as skin conductance response and other relevant parameters for measuring subjective stress [12]–[14] (Vicario et al., 2020a; Ney et al., 2021; Marković et al., 2021) in response to VR fear relevant scenarios, are processed by artificial intelligence in terms of the patient's biofeedback signals to recognize and establish, in real time, the correct level of (virtual) exposure therapy to the source of fear. This could be useful to patients as it would consent the autonomous fear management in the home setting, and to the psychotherapists, as it would allow a remote control/supervision of the effectiveness of the intervention program [11] (Lucifora et al., 2021b). Moreover, VR can be combined with mindfulness, an effective practice to improve clinical symptoms [15]–[18] (Feruglio et al., 2021; Conversano et al., 2020; Di Giuseppe et al., 2019; 2022), including hypertension [19] (Conversano et al., 2021). Evidence suggests that mindfulness is more effective when combined with VR. A recent review found that VR-based mindfulness training is more efficient at reducing anxiety and depression, and improving sleep quality, emotion regulation, and mood, compared to standard mindfulness protocols [20] (Ma et al., 2022). Finally, promising therapeutic applications can derive from the combination of VR with non-invasive brain stimulation (NIBS) methods, which effectiveness in the clinical field is well documented [21]–[27] (e.g., Vicario and Nitsche, 2013a, 2013b; Rivera-Urbina et al., 2017; Vicario et al., 2020b; Salehinejad et al., 2020; Salehinejad et al., 2021; Anselmo et al., 2022). This is suggested by the study of van 't Wout-Frank et al. (2019) [28] documenting a clinically meaningful reduction in symptoms severity in a group of veterans with as post-traumatic stress disorder (PTSD) after two weeks of transcranial direct current stimulation of the left ventromedial prefrontal cortex received during simultaneous exposure to war combat-related VR scenarios.

Insights supporting the importance and efficacy of VR in the clinical field is also provided by research on the rehabilitation of neurological disorders. For example, a recent review article investigating the relevance of VR in stroke rehabilitation found that this technology can be more effective than standard therapies in improving upper limb function and activities of daily living function when used in addition to usual care [29] (Laver et al., 2017). Moreover, there is evidence that VR based trainings are effective in slowing the effects of neurodegeneration in Alzheimer's disease. In a single case study [30] (White et al., 2016), a man at the onset of Alzheimer's disease (AD) was enrolled in a cognitive treatment program based upon spatial navigation in VR environment. The patient learned to navigate towards the desired targets in the VR environment. Moreover, according to the primary caregiver's report, VR training has provided significant benefits in managing activities and routines of daily living.

Finally, VR can be useful for creating experimental settings relevant for the study of clinical symptoms through the involvement of healthy participants. This is the case of Pavlovian conditioning protocols (Lucifora et al., 2022) [31] where VR is used to create relevant scenario for the study of fear learning and extinction, two mechanisms crucially involved in anxiety and related disorders (PTSD) [32],[33] (Vicario et al., 2019; Vicario et al., 2022).

In conclusion, the current state of the art suggests that the use of VR technology has great potential in boosting clinical practice by promoting therapeutic efficacy, especially when combined with other well established therapeutic methods such as mindfulness and NIBS. The community of clinical psychologists and clinical neuroscientists should look with enthusiasm and genuine interest in VR technology as it could make a difference in psychotherapy and neurorehabilitation.
